# Efficacy of Formulation With Potential as Herbal Medicine on Second Degree Burn Wound: Biochemical and Molecular Evaluation

**DOI:** 10.1111/jocd.70122

**Published:** 2025-03-18

**Authors:** Seda Askin, Merve Kaynarpinar

**Affiliations:** ^1^ Health Services Vocational School Ataturk University Erzurum Turkey; ^2^ Institute of Health Sciences, Ataturk University Erzurum Turkey

**Keywords:** anti‐inflammatory, burn wound, cytokines, growth factors, healing, qPCR

## Abstract

**Background:**

Burn injury is a condition caused by heat, cold, electricity, synthetic substances, and radiation, and it causes psychological and physical problems in the affected individuals.

**Aims:**

In this study, it was aimed to investigate the healing effect of the spray formulation prepared using ethanol extracts of *Olea europaea* and *Aloe vera* leaves, *Cocus nucifera* fruit, and *Chamomilla recutita* flower plants (OACC) in a second‐degree burn model created in rats, using biochemical and molecular parameters.

**Methods:**

Experimental groups were assigned to Healthy control (HC), Burn control (BC), Silver‐Sulfadiazine (SS) and OACC. A deep second‐degree burn was induced on the lower back and upper back of each rat under standard burning procedures, respectively. Experiments were performed using serum and skin tissue samples obtained on the 3rd–21st days after the burns were created. Malondialdehyde (MDA) and superoxide dismutase (SOD) levels were calculated. Transforming Growth Factor Beta‐1 (Tgf‐β1), Vascular Endothelial Growth Factor‐alfa (Vegf‐α), interleukin‐6 (Il‐6) and Tumor Necrosis Factor Alpha (Tnf‐α) mRNA expression levels were determined using real‐time polymerase chain reaction (RT‐PCR).

**Results:**

AOCC reduced the increased MDA levels in serum related to the burning event, while increasing the decreased SOD enzyme activity levels. In addition, AOCC decreased the gene expression levels of Tgf‐β1 and Vegf‐α, which are growth factors that were increased in the burn group, and Il‐6 and Tnf‐α, which are oxidative stress markers.

**Conclusions:**

We believe that our study will shed light on the detailed examination of biochemical and molecular pathways affecting the wound healing process in future studies and will contribute to opening new doors for treatment.

## 1 Introduction

Burn injuries, which are much more common in developing or underdeveloped countries, continue to be a major public health problem [1]. When classified within themselves, flame burns, scalding, hot water, and steam are the most common [2]. Burn injuries are a complex condition, and the most important factors are the depth of the burn, the anatomical location of the area, and the occurrence of infection. For these reasons, although a wide variety of approaches are recommended for burn treatments, healing with modern treatments is still a difficult process [3]. Considering the major problems experienced today in terms of the cost of drugs and the development of allergies and drug resistance, medicinal plants used in traditional methods and having wound healing effects have proven to be more affordable, effective, and reliable [4]. Plants are used in many parts of the world for wound and burn treatment and are known to be very effective [5, 6].

As a result of in vitro and in vivo studies conducted with these plants, it has been confirmed that they can be used in burn and wound treatment. Among these traditional medicinal plants, *Aloe vera* and *Olea europaea* leaves, *Cocus nucifera* fruit, and *Chamomilla recutita* flower were the main characters of the presented study. It has been reported that *Aloe vera* restarts angiogenesis, accelerates blood flow, increases fibroblast proliferation, and has anti‐inflammatory and antimicrobial effects [7]. *Olea europaea* has antioxidant, anti‐inflammatory, antiviral, and antifungal effects thanks to the phenolic compounds it contains [8]. In addition to its antiviral and antimicrobial properties, *Cocus nucifera* has also been found to contain high amounts of tocopherols [9]. Finally, it is known that *Chamomilla recutita* is important for macrophages involved in the pathogenesis of many diseases such as infections and neuropsychiatric problems [10]. In this study, the healing effect of *Aloe vera* and *Olea europaea* leaf, *Cocus nucifera* fruit, and *Chamomilla recutita* flower mixed extract (AOCC) spray formulation on burn wounds in a second‐degree burn model created in rats was investigated using biochemical and molecular parameters. For this purpose, MDA and SOD levels were determined to evaluate the antioxidant properties of AOCC. In addition, mRNA expressions of Tgf‐β1, Vegf‐α, Il‐6, and Tnf‐α genes, which are the main genes that regulate inflammation, angiogenesis, and oxidative stress processes, which are known mechanisms that affect wound healing and play a role in these pathways, were analyzed.

## 2 Materials and Methods

### 2.1 Preparation of AOCC Mixture Spray Form

Spray formulation was prepared using ethanol extracts of *Olea europaea* and *Aloe vera* leaves, *Cocus nucifera* fruit, and *Chamomilla recutita* flower plants. Each powdered plant material was weighed equally at 30 g, and each was transferred to a separate conical flask. 1500 mL of ethanol was added to each conical flask; the mouths were tightly closed with paraffin and left to wait for 72 h for the active ingredients to pass into the solvent. At the end of the period, the solution in the conical flask was filtered with a filter paper. Then, it was put in a rotavapor (47°C) until there was no ethanol left. The plant extracts prepared differently were transferred to a common volumetric flask, and glycerin was added. The prepared extract was filled into a dark bottle, sprayed with a spray head, and stored at +4°C [11].

### 2.2 Animals, Experimental Design and Second‐Degree Burn Induction

**FIGURE 1:**
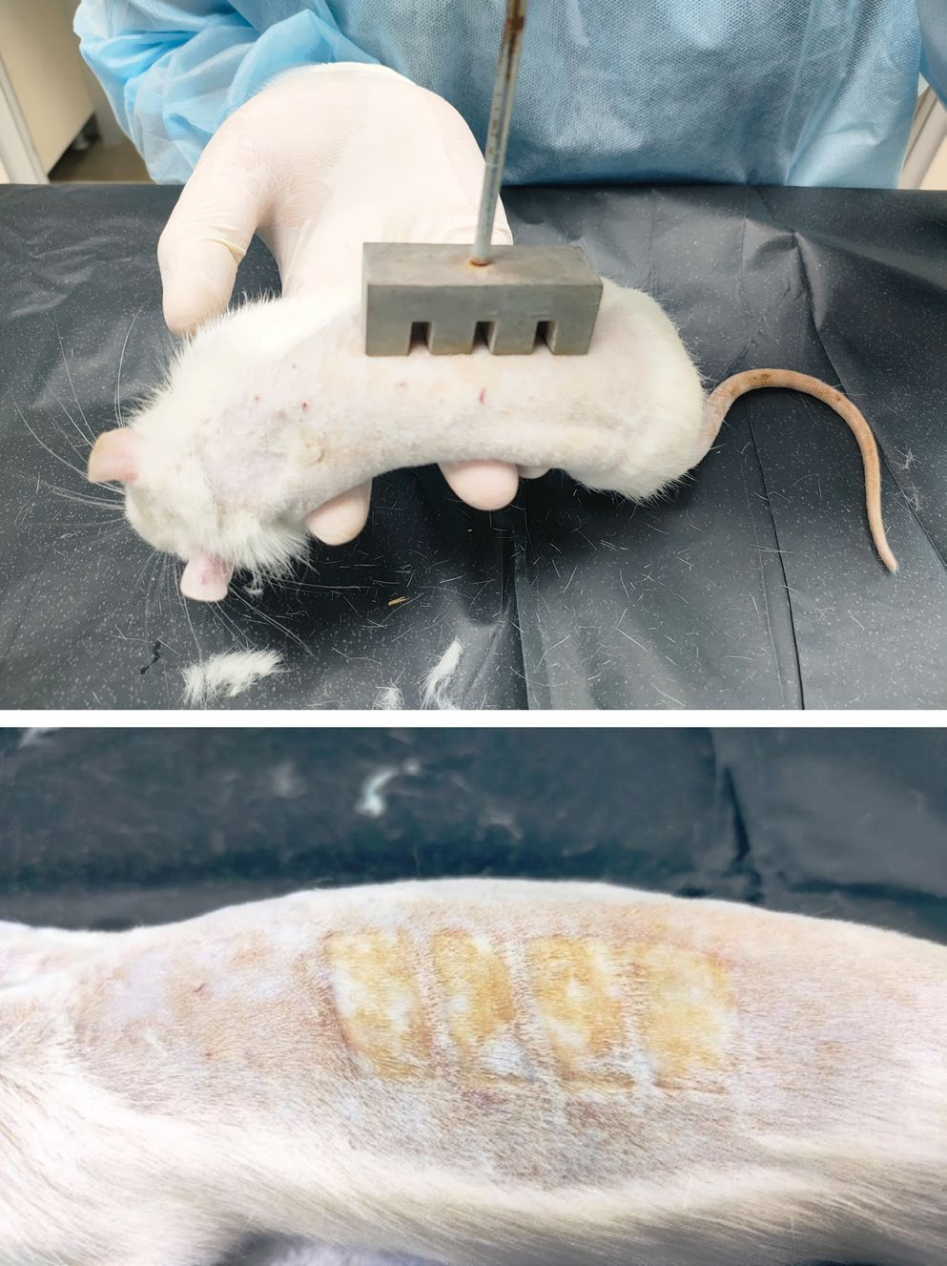
Creation and appearance of a second degree burn.

This study was conducted after the ethical approval of the institution with which we are affiliated. Applications were made on 48 Wistar albino male rats (280–375g). Before the experiment started, the rats were kept in polycarbonate cages at room temperature (22°C ± 2°C) in groups of 6, with free access to water and food. The cages were kept in a 12‐h light and 12‐h dark environment [12]. All rats were divided into four groups according to their similar weights: (A) Healthy control (HC) group (*n* = 12); (B) Burn control (BC) group (*n* = 12); (C) Silver Silfadiazine (SS) group (*n* = 12); (D) Spray treatment (AOCC) group (*n* = 12). In order to be able to apply immediately after shaving, the metal blocks were kept in water heated to 95°C with thermometer monitoring for a few minutes. The metal blocks heated in this way were applied to the anesthetized animals for 20 s, and burns were created on the right and left backs (Figure 1).

Rats were placed in experimental groups with their weights close to each other. No other procedure was performed on the healthy group animals except shaving. Burn control group animals were only burned, and no treatment was applied. The animals in the Silver Silfadiazine group were treated with Silverdin Cream (Silver Sulfadiazine Cream 1%, Deva) (40 g/kg) once a day to cover the wound surface. The AOCC spray mixture was sprayed on the AOCC spray treatment group animals once a day from a distance of 10 cm to cover the wound areas sufficiently [13]. Treatment applications were started 12 h after the burns were created. Three animals were sacrificed randomly from each group on the 3rd, 7th, 14th, and 21st days of the experiment. Skin and blood samples were taken from the sacrificed animals. Necessary procedures were continued for the remaining group animals. Digital photographs were taken on days 0, 3, 7, 14, and 21. The burn treatment was evaluated using Image J software (NIH, USA) to record the wound areas along with the wound changes before and after [1].

### 2.3 Preparation and Storage of Serum and Tissue Samples

On the 3rd, 7th, 14th, and 21st days of the experiment, the animals selected were anesthetized, and blood samples were taken into heparinized tubes by entering the left ventricle of the heart with a syringe [14, 15]. All blood samples were centrifuged at 4000 rpm for 10 min. The obtained sera were stored in a −80°C freezer for biochemical analyses. In addition, for molecular studies, burnt skin samples were taken from the right and left dorsal regions, surrounded by healthy skin, and frozen with liquid nitrogen and then stored at −80°C. For the homogenate process, the skin tissues at −80°C were brought to +4°C and kept for one day. 0.3 mg of sections were taken from each tissue, and the macro disintegration process was performed. Then the tissues were placed in screw‐capped tubes, and 1 mL (0.1M KH2PO4‐10Mm EDTA, Ph: 7) of homogenization buffer and steel homogenate beads were added. The tubes were homogenized in the TissueLyser LT (Qiagen) homogenizer device with five repetitions at 3000 rpm. After centrifugation, the supernatant portions were taken into 1.5 mL Eppendorfs, and the pellet portions were removed.

### 2.4 Biochemical Studies

Biochemical studies carried out within the scope of the presented study included the amount of malondialdehyde (MDA) for the determination of lipid peroxidation and superoxide dismutase (SOD) for the determination of antioxidant enzymes [16].

Malondialdehyde (MDA) levels were determined using the thiobarbituric acid (TBA) reaction in serum samples obtained from rats. Homogenized serum samples (125 μL) were added to TBS (50 μL, pH: 7.4) and TCA‐BHT (125 μL). After vortexing and centrifuging (1000 rpm/10 min), 200 μL of the supernatant of the resulting mixture was taken and added to HCl (40 μL, 0.6 M) and Tris‐TBA (160 μL). Then, it was incubated at 80°C/10 min. TBS was considered as blank, and absorbance values were measured in the spectrophotometer (530 nm). Experiments were performed in triplicate. TBARS concentration was determined according to Guesmi et al. [17] was estimated according to the formula used. The measurement of SOD enzyme activity is based on the reduction of the nitroblue tetrazolium (NBT) compound as a result of a reaction in which the superoxide radical generated by the xanthine‐xanthine oxidase system cannot be removed by the SOD enzyme. Briefly, 10 μL xanthine oxidase, 200 μL assay reagent, 10 μL samples and 40 μL distilled water were added to each well. It was incubated at 25°C for 20 min. After 20 min, the absorbance of the colored complex formed was read at a 560 nm wavelength by spectrophotometric methods [18].

### 2.5 Molecular Studies

Within the scope of the study, total RNA extraction, cDNA synthesis, and real‐time quantitative PCR analyses were performed in skin tissue samples, and Tnf‐α, IL‐6, Tgf β1 and Vegf‐α gene expression levels were calculated. The tissues obtained as a result of the applications were obtained in a homogenized form using a homogenizer. RNA isolation was performed using the RNeasy Mini Kit (Qiagen). Then, cDNA was obtained from these RNA samples with the High Capacity cDNA Reverse Transcription Kit. cDNA concentration and quality were assessed and quantified according to the method used by Avsar et al. [14]. Primers for Tgf‐β1, Vegf‐α, IL‐6, Tnf‐α and housekeeping gene Gapdh were used and performed with the Real‐time PCR (Bio‐Rad) kit. First, the required Master mix was created, and 18 μL was added to each tube. Samples were prepared by adding 2 μL of cDNA samples to the tubes to which the Master mix was added. Then, the prepared samples were loaded into the Rotor‐Gene PCR device, and their cycles were set. All steps of this process were applied separately for each gene, and the results were analyzed.

### 2.6 Statistical Evaluation

Analysis of Variance (ANOVA) was applied for statistical evaluation of the data obtained as a result of the experiments. Differences between groups were determined by the Tukey test. The Duncan test was preferred for mean differences between groups. GraphPadPrismversion10 was used to test quantitative real‐time PCR results. The mean of the data was given as ± standard deviation. *p* values were determined using an unpaired *t* test. Significance level ****p < 0.0001 was evaluated as very significant, ***p < 0.0001 as very significant, **p < 0.0001 as significant, and *p* > 0.0001 as insignificant.

## 3 Results

### 3.1 Visual Assessment of Wound Dimensions

**FIGURE 2:**
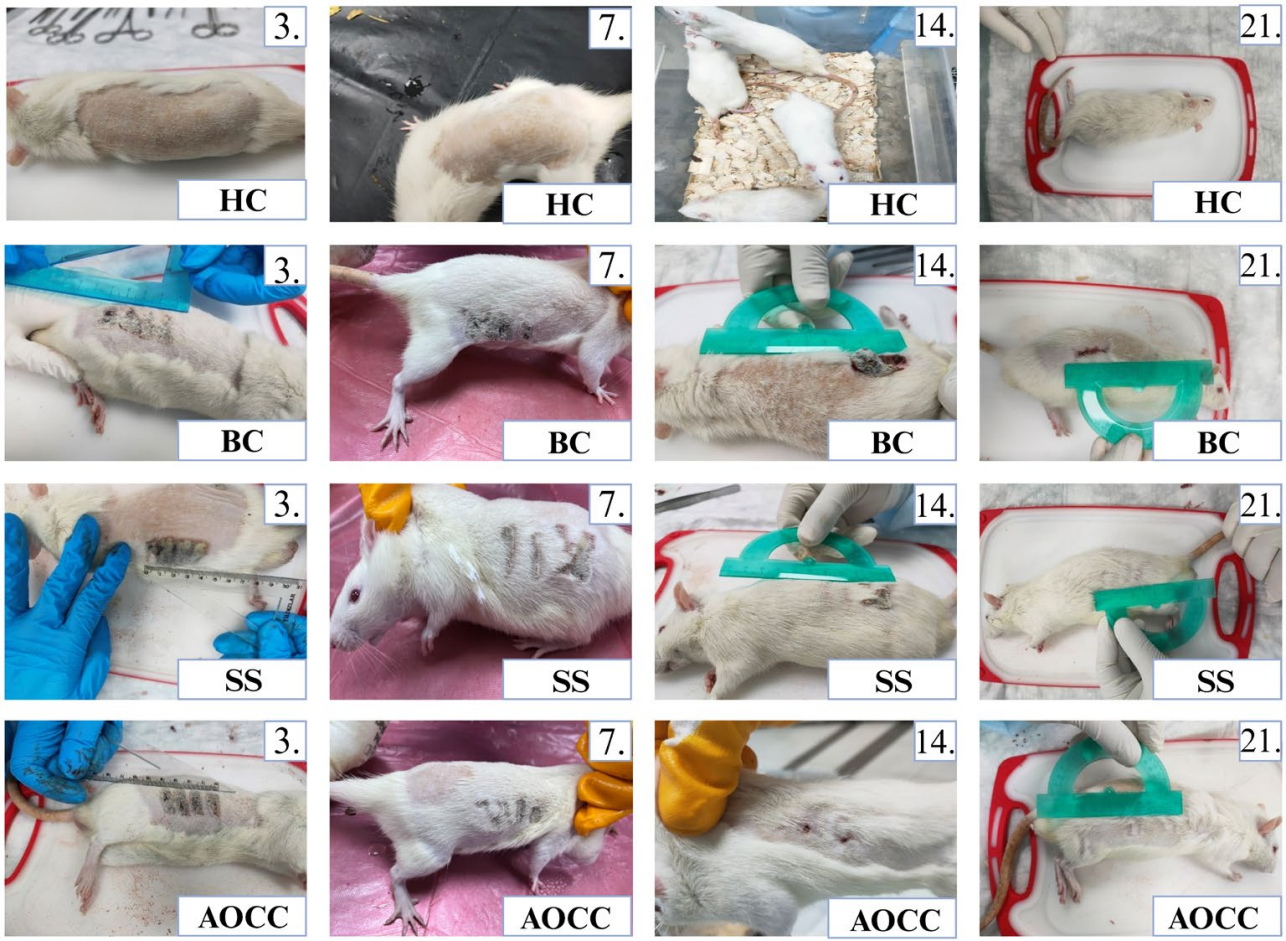
Images of application groups on different days.

Wound size and healing rate were monitored during the experimental process. Images of animals photographed on different days from all application groups are shown in Figure 2. On the 3rd day of the experimental process, all application groups were similar to each other. No significant difference was observed visually between them. On the 7th day of the experiment, reductions in wound size and more pronounced wound areas were observed in the other application groups compared to the BC group. The wound image in the AOCC group showed the same healing effect as the SS group, which is widely used in burn treatment. On the 14th day of the applications, it was observed that the wound size was reduced and hair growth was faster, especially in the AOCC group compared to the BC group. It was observed that wound healing in the AOCC was also faster than in the SS group. Finally, on the 21st day, the last day of the experiment, it was observed that skin epithelization was completely completed in the experimental animals in the AOCC group and the wound site was completely closed. While a similar situation was observed in the experimental animals in the SS group, it was determined that the wound had not yet closed in the BC group animals (Figure 2).

### 3.2 MDA and SOD Values From Oxidative Stress Parameters

In the presented study, the results of oxidative stress parameters (MDA and SOD) in serum samples obtained from the treatment groups are given in Table 1 and Table 2. As indicated in Table 1, serum samples from the untreated burn‐induced group (BC) showed higher TBARS levels on all application days compared to the healthy non‐burned control group (HC). In the HC group, the 21‐day TBARS concentration change was measured between 46.171 ± 3.04 nmol/mg and 67.792 ± 2.06 nmol/mg (day 3 and day 21). In the BC group, these values were measured between 234.684 ± 4.79 nmol/mg and 260.360 ± 3.72 nmol/mg, respectively. Treatment with SS and AOCC significantly reduced serum lipid peroxide levels as measured by TBARS concentration (229.504 ± 8.11 nmol/mg to 155.630 ± 12.84 nmol/mg for days 3 and 21, respectively; 227.477 ± 11.90 nmol/mg to 121.396 ± 9.77 nmol/mg for days 3 and 21, respectively), approaching the HC group. When the AOCC and SS groups were compared, it was found that AOCC reduced lipid peroxidation much more strongly.

**TABLE 1:** Multiple comparison test results of MDA levels and group mean data.

Groups	3.day (nmol/mg)	7.day (nmol/mg)	14.day (nmol/mg)	21.day (nmol/mg)
HC	46.171 ± 3.04^a^ *	51.351 ± 8.10^a^ *	65.540 ± 3.76^a^ *	67.792 ± 2.06^a^ *
BC	234.684 ± 4.79^b^	247.972 ± 2.02^d^	254.729 ± 7.30^d^	260.360 ± 3.72^b^
SS	229.504 ± 8.11^b^	215.089 ± 5.62^c^	190.990 ± 5.41^c^	155.630 ± 12.84^c^
AOCC	227.477 ± 11.90^b^	191.891 ± 7.67^b^	151.126 ± 7.83^b^	121.396 ± 9.77^b^

Abbreviations: AOCC, spray treatment; BC, burn control; HC, healthy control; SS, silver sulfadiazine.

* The difference between the application groups indicated with different symbols is significant (*p* < 0.05), while the difference between the application groups indicated with the same symbol is insignificant (*p* > 0.05).

**TABLE 2:** Multiple comparison test results of SOD levels and group mean data.

Groups	3.day (nmol/mg)	7.day (nmol/mg)	14.day (nmol/mg)	21.day (nmol/mg)
HC	11.166 ± 3.93^b^	9.466 ± 6.46^b^	9.792 ± 7.17^b^	10.694 ± 7.17^b^
BC	8.976 ± 4.52^a^ *	7.387 ± 6.08^a^ *	5.583 ± 7.87^a^ *	3.092 ± 5.61^a^ *
SS	15.332 ± 3.64^c^	16.299 ± 10.02^c^	19.885 ± 8.57^c^	23.501 ± 9.66^c^
AOCC	15.525 ± 4.55^c^	19.069 ± 2.57^d^	23.278 ± 9.04^d^	30.837 ± 10.33^d^

Abbreviations: AOCC, spray treatment; BC, burn control; HC, healthy control; SS, silver sulfadiazine.

* The difference between the application groups indicated with different symbols is significant (*p* < 0.05), while the difference between the application groups indicated with the same symbol is insignificant (*p* > 0.05).

When the SOD levels measured in the serum samples on the 3rd, 7th, 14th, and 21st days were examined, SOD values in the BC group decreased compared to the HC group on all days. In the SS and AOCC treatment groups, the decreases in SOD levels caused by burns were replaced by high SOD levels. As follows; the SOD value, which was 8.976 ± 4.52 nmol/mg on the 3rd day in the BC group, decreased to 3.092 ± 5.61 nmol/mg on the 21st day. In the SS group, these values were 15.332 ± 3.64 nmol/mg and 23.501 ± 9.66 nmol/mg, respectively. The most striking increases were again in the AOCC application group. The SOD value measured on the 21st day was 30.837 ± 10.33 nmol/mg (Table 2).

### 3.3 Gene Expression Levels (Tgf‐β1, Vegf‐α, IL‐6, and Tnf‐α)

**FIGURE 3:**
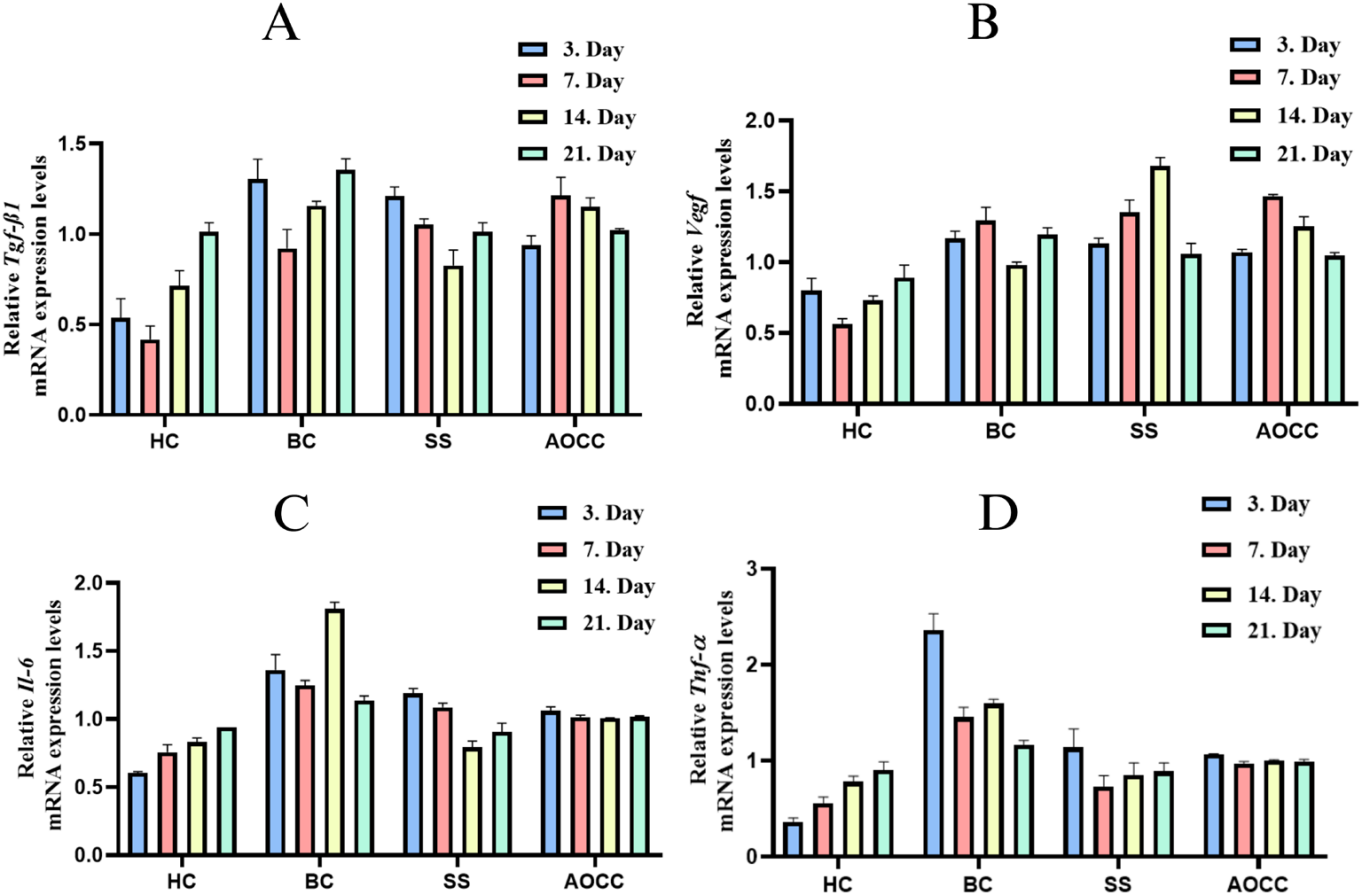
Gene expression results of related genes in skin tissues (AOCC, spray treatment group; BC, burn control group; HC, healthy control group; SS, silver silfadiazine group).

Data on relevant gene expression levels (Tgf‐β1, Vegf‐α, IL‐6, and Tnf‐α) are given in Figure 3. As shown in Figure 3A, the Tgf‐β1 gene expression was increased in all burn groups (BC 3d, 7d, 14d and 21d) by 2.43‐ fold, 2.21‐fold, 1.62‐fold, and 1.34‐fold, respectively, compared to the HC group (*p* < 0.05). In SS treatment groups, Tgf‐β1 gene expression increased 2.26‐fold, 2.53‐fold, and 1.16‐fold in SS 3d, 7d, and 14d groups, respectively, compared to the HC group and decreased 1.38‐fold in the SS 21d group (*p* < 0.05). AOCC treatment increased Tgf‐β1 mRNA expression in all AOCC groups (AOCC 3d, 7d, 14d and 21d) by 1.77‐fold, 2.92‐fold, 1.61‐fold, and 1.01‐fold, respectively, compared to the HC group as shown in Figure 3A (*p* < 0.05). Tgf‐β1 levels increased on day 3 in the BC group but were lower in the SS and AOCC treatment groups. On day 7, Tgf‐β1 levels were higher in the treatment groups. On day 14, Tgf‐β1 levels were high in the BC group but decreased especially in the SS treatment group. On day 21, Tgf‐β1 levels were highest in the BC group, while they decreased in the SS and AOCC treatment groups and were measured close to each other.

In the BC group treatment group rats, the Vegf‐α mRNA gene expressions of all days also showed a significant increase, as well as in the expression levels of other genes, when compared with the HC group data. These increases ranged from 1.34 to 2.33 fold. In the SS group treatment group rats, the Vegf‐α mRNA gene expressions of all days also increased compared to the HC group. The increases ranged from 1.19 to 2.42 fold (*p* < 0.05). In the AOCC treatment group rats, the Vegf‐α mRNA gene expression levels of all days were significantly increased compared to the HC group: 1.34 fold, 2.64 fold, 1.71 fold, and 1.18 fold, respectively (*p* < 0.05).

On day 3, Vegf‐α levels were high in the BC group, while they tended to decrease slightly in the SS and AOCC treatment groups. On day 7, Vegf‐α levels tended to increase slightly in the treatment groups compared to the BC group. On day 14, Vegf‐α levels were again quite high in the treatment groups compared to the BC group (especially in the SS group). On day 21, a decrease in Vegf‐α levels was observed in the SS and AOCC groups compared to the BC group, but there was no significant difference between the treatment groups, as shown in Figure 3B (*p* < 0.05).

As shown in Figure 3C, the IL‐6 gene expression was increased in all burn groups (BC 3d, 7d, 14d and 21d) by 2.26‐fold, 1.65‐fold, 2.18‐fold, and 1.21‐fold, respectively, compared to the HC group (*p* < 0.05). In SS treatment groups, IL‐6 gene expression increased 1.96‐fold and 1.44‐fold in SS 3d and 7d, respectively, compared to the HC group, and decreased 1.05‐fold and 1.04‐fold in SS 14d and 21d groups (*p* < 0.05). AOCC treatment significantly increased the IL‐6 gene expressions by 1.76‐fold, 1.34‐fold, 1.21‐fold, and 1.08‐fold, respectively, for AOCC 3d, 7d, 14d, and 21d groups compared to the HC group (*p* < 0.05). While the IL‐6 gene levels were high in the BC group on the 3rd and 7th day data, these values were lower in the SS and AOCC treatment groups. On the 14th day, the BC IL‐6 value was the highest, and the lowest in the SS group. On the 21st day, the IL‐6 values obtained from the BC, SS, and AOCC groups were found to be very close to each other.

Similarly, Tnf‐α mRNA gene expression increased (Figure 3D) in the BC 3d, 7d, 14d, and 21d group rats compared to the HC group (6.56‐fold, 2.59‐fold, 2.06‐fold and 1.29‐fold, respectively). In SS treatment groups, Tnf‐α gene expression increased 3.16‐fold, 1.31‐fold, and 1.08‐fold in SS 3d, 7d, and 14d groups, respectively, compared to the HC group, and decreased 1.02‐fold in the SS 21d group (*p* < 0.05). AOCC treatment significantly increased the Tnf‐α gene expressions by 2.97‐fold, 1.73‐fold, 1.28‐fold, and 1.09‐fold, respectively, for AOCC 3d, 7d, 14d, and 21d groups compared to the HC group (*p* < 0.05). The Tnf‐α gene level showed its highest value in the BC group on day 3. The lowest Tnf‐α gene level was seen in the SS group on day 7. The Tnf‐α gene levels of the AOCC treatment group were at similar levels on all days.

## 4 Discussion

Burn wound repair is a complex process that includes hemostasis, vascularization of the burn skin, inflammation, re‐epithelialization, and tissue remodeling by restoring collagen production. Due to its prevalence and high frequency in society today, the development of therapeutic treatments for skin wound repair has become a priority for scientists [19]. Topical drugs that are frequently used for this repair and have a widespread marketing area worldwide are usually produced with antibiotic, antimicrobial, and antiseptic active ingredients. In addition, research on the therapeutic effects of the direct use of herbal cures, especially herbal mixtures and extracts, which increase epithelial formation and have antioxidant properties, has been rapidly increasing recently. In recent years, researchers have been developing more useful and technological methods such as biomaterials, biogels, and sprays prepared with effector materials to clean wounds quickly and painlessly and to maintain wound integrity and moisture [20]. In our study, we investigated the healing effects of the spray form of the mixture of *Aloe vera, Olea europaea, Chamomilla recutita*, and *Cocos nucifera* (AOCC) plant extracts, which have not been investigated as a mixture before, in a second‐degree burn model. We demonstrated the therapeutic effects of AOCC in terms of oxidative stress markers and the expression levels of genes that regulate oxidative stress processes and inflammation in wound healing.

MDA is a metabolite of lipid peroxidation, which is also an indicator of membrane stability in cells and one of the important indicators of the presence of oxidative stress in cells and tissues [21]. Similarly, the parameter used to determine antioxidant mechanisms in tissues is the measurement of SOD enzyme values. Increased MDA levels and decreased SOD levels in tissues are considered indicators of oxidative stress [22]. In our study, MDA and SOD parameters were selected because they are important indicators in reflecting redox balance, along with their known functions in oxidative homeostasis for the cell and their potential therapeutic effects [23]. In our study, parallel to the literature, when the MDA and SOD levels obtained from the BC group individuals were compared with the results obtained from the HC group individuals, it was found that the MDA levels in the BC group individuals increased significantly on the 3rd, 7th, 14th, and 21st days, while the SOD levels decreased significantly (*p* < 0.05). In the SS and AOCC groups, MDA levels decreased significantly on the 3rd, 7th, 14th, and 21st days, while SOD levels increased significantly (*p* < 0.05). In a study examining the healing, rejuvenating, and scar‐healing effects of mesenchymal stem cells and platelet‐rich cells on skin burns, it was reported that SOD activities decreased in burn control groups, while MDA levels increased significantly. In the same study, in contrast to this situation, SOD activities increased in burnt tissue samples in the treatment groups, while MDA levels decreased [24].

One of the most important mechanisms that provide regeneration in the wound healing process is growth factors [20]. It is well known that growth factors play a role in cell proliferation, angiogenesis, which means the formation of new vessels by budding from existing vessels, and the formation of new connective tissue (granulation tissue) containing microscopic blood vessels and myofibroblasts that develop in the wound area during the healing process, and their importance in the wound healing process [25]. In the current study, we analyzed Tgf‐β1 and Vegf‐α. Tgf‐β1 plays important roles in the wound healing center [20, 25]. It is now well known that Tgf‐β1 stimulates myofibroblast differentiation, which is a feature of fibrotic diseases [26]. Tgf‐β1 also stimulates some cells that are important components of wound healing mechanisms, including monocytes, endothelial cells, keratinocytes, and fibroblasts [27]. Finally, it is emphasized that Tgf‐β1 is a type of cytokine that regulates functional events such as cell proliferation, cell motility (diapedesis), cellular differentiation, and cellular adhesion [28]. In a study conducted by Schultze‐Mosgau et al. [29], inhibition of Tgf‐β1 activity showed less fibrosis and significant collagen type I–IV fiber production. Therefore, Tgf‐β1 may be the driving force behind new connective tissue formation. Ghahary and colleagues reported that healthy skin formation has higher Tgf‐β mRNA levels than burned skin [30].

In parallel with these results, AOCC treatment induced fibroblast activity, epithelial regeneration, and keratinization by increasing Tgf‐β1 levels. In contrast, there is an opposite result where Tgf‐β1 delays re‐epithelialization, and Tgf‐β1 inhibition promotes wound healing. However, in the same study, Tgf‐β1 inhibition was shown to reduce fibrosis and myofibroblast differentiation during the wound healing process [31]. The reason for these conflicting results is probably due to the tissue samples studied belonging to different tissues because they studied corneal wound healing while we studied skin wound healing.

Like the Tgf‐β1, Vegf‐α plays many roles in angiogenesis, which is important in the skin regeneration process [32]. Li et al. [33] showed that Vegf‐α mRNA expressions decreased in second‐degree burnt skin [33]. In a similar study, Bayir et al. [20] showed that Vegf‐α expression values decreased in the burnt control group. These researchers showed that the mixture of beeswax, olive oil, and butter they used for treatment significantly increased the Vegf‐α mRNA expression level. In our current study, we showed that Vegf‐α levels decreased in burnt skin, while AOCC treatment increased Vegf‐α expressions. It was thought that AOCC treatment increased Vegf‐α levels and provided new vessel formation.

Pain, redness, swelling, and inflammation, which are considered natural responses of cells to injury and infection and are necessary for cell homeostasis, are very important elements for the tissue to regain its former state and to re‐function [34]. Local cells, intracellular mediators, and intercellular matrix elements play a role in the inflammatory process. With a beneficial inflammatory response for cells, agents that damage cells and tissues are cleared, and physiological functioning is restored. Since an unsuccessful inflammatory response will cause morbidity, it can have negative effects on many vital pathways in cells. Also, excessive inflammation and weakened immune response can damage normal tissues and prevent wound closure, becoming the primary causes of abnormal wound formation [35]. In these cases, cytokines are activated. In particular, IL‐6 and Tnf‐α are the most important of these cytokines. Detection of expression levels of proinflammatory cytokines such as Tnf‐α, IL‐6, and IL‐1β in serum or tissue is among the frequently preferred markers in wound healing studies [36].

In the present study, we also studied mRNA gene expression levels of IL‐6 and Tnf‐α genes, which are closely related to growth factors. In our study, as a result of burn application, the level of IL‐6 gene expression increased significantly, as expected. This increase created an inflammatory response in the skin tissue, leading to the accumulation of inflammatory‐related cells and distant organ damage. On the contrary, AOCC treatment reversed the significant increase in IL‐6 levels triggered by burn and brought them to the healthy control level. When our results for another inflammatory marker, Tnf‐α, were evaluated for AOCC, the results were similar to the IL‐6 results.

Especially the IL‐6 gene has great effects on the pathogenesis of inflammatory diseases in terms of its effect on the innate or acquired immune system and systemic effects. Therefore, IL‐6 has become a target in treatments. It plays a role in gene activation and the initiation of a wide range of biological activities in the cell by binding to the cell membrane. In addition, the IL‐6 gene increases the activation of neutrophils, attracts marginal neutrophils to the circulation, and causes an increase in Vegf levels in synovial cells by creating a synergistic effect with Tnf‐α. In this way, it is responsible for angiogenesis and endothelial cell proliferation with a chain of interactions [37].

It has been determined by studies conducted on diabetic rats that the polyphenols in the olive leaf extract contained in AOCC have antioxidant activity, affect, and facilitate the wound healing process, and olive leaves also have antibacterial activity [38]. It has also been determined that the extract obtained from Aloe vera leaves contained in the AOCC causes the emergence of hair follicles in the skin and an increase in the skin's natural epithelial cells; thus, the cells begin to return to their normal activity with an increase in the collagen rate [39]. The study of extracts obtained from coconut husk fibers and fruit revealed that these extracts exhibited antimicrobial activity against Staphylococcus aureus, a common cause of skin infections [40]. It has been reported that *Chamomilla recutita* (*Matricaria recutita*) can be evaluated as a potential candidate in the design of effective antifungal formulations suitable for the treatment of dermatophytosis and other fungal infections. These nutritious and infection‐protecting products provide benefits in preserving the moisture of the skin as well as their ability to renew the skin layers [41].

## 5 Conclusions

In this study, the medical value of the application of AOCC spray mixture in the wound healing of deep second‐degree burns in Wistar rats was investigated at biochemical and molecular levels. For this purpose, changes in MDA and SOD biochemical parameters and Tgf‐β1, Vegf‐α, IL‐6, and Tnf‐α gene expression levels were examined. It can be said that the AOCC spray mixture exhibits positive effects on burn‐inducing rats thanks to the ingredients in its content. These positive effects of AOCC can be attributed to its antioxidant, antiseptic, and anti‐inflammatory properties. We believe that our study will shed light on the detailed examination of biochemical and metabolic pathways affecting the wound healing process in future research and that AOCC obtained by traditional methods may be an effective wound care product.

## Conflicts of Interest

The authors declare no conflicts of interest.

## Data Availability

The data that support the findings of this study are available from the corresponding author upon reasonable request.

## References

[1] K. Wang , K. Shen , F. Han , et al., “Activation of Sestrin2 Accelerates Deep Second‐Degree Burn Wound Healing Through PI3K/AKT Pathway,” Archives of Biochemistry and Biophysics 743 (2023): 109645, 10.1016/j.abb.2023.109645. 37225009

[2] A. M. Al‐Mutairi , S. Labani , M. J. Alasmari , et al., “Burn Injury Characteristics and Outcomes Among Pediatric and Adult Patients Admitted to Ministry of National Guard Health Affairs (MNGHA) Hospitals in Saudi Arabia,” Burns Open 7, no. 4 (2023): 146–152, 10.1016/j.burnso.2023.09.002.

[3] A. Oryan , E. Alemzadeh , and A. Moshiri , “Burn Wound Healing: Present Concepts, Treatment Strategies and Future Directions,” Journal of Wound Care 26, no. 1 (2017): 5–19, 10.12968/jowc.2017.26.1.5. 28103165

[4] Z. Tessema and Y. Molla , “Evaluation of the Wound Healing Activity of the Crude Extract of Root Bark of Brucea Antidysentrica, the Leaves of Dodonaea Angustifolia and Rhamnus Prinoides in Mice,” Heliyon 7, no. 1 (2021): e05901, 10.1016/j.heliyon.2021.e05901. 33521349 PMC7820475

[5] İ. Dursun , R. Sağlamtaş , K. Fettahoğlu , et al., “Antioxidant and Antimicrobial Activities of Different Extracts of Tragopogon Dubius and Tragopogon Porrifolium L. Subsp. Longirostris: Determination of Their Phytochemical Contents by UHPLC‐Orbitrap®‐HRMS Analysis,” Food Bioscience 63 (2025): 105604.

[6] M. S. Karağaç , E. N. Yeşilkent , D. Kizir , et al., “Esculetin Improves Inflammation of the Kidney via Gene Expression Against Doxorubicin‐Induced Nephrotoxicity in Rats: In Vivo and In Silico Studies,” Food Bioscience 62 (2024): 105159.

[7] P. Farzadinia , N. Jofreh , S. Khatamsaz , et al., “Anti‐Inflammatory and Wound Healing Activities of Aloe Vera, Honey and Milk Ointment on Second‐Degree Burns in Rats,” International Journal of Lower Extremity Wounds 15, no. 3 (2016): 241–247, 10.1177/1534734616645031. 27217089

[8] T. Toulabi , B. Delfan , M. Rashidipour , et al., “The Efficacy of Olive Leaf Extract on Healing Herpes Simplex Virus Labialis: A Randomized Double‐Blind Study,” EXPLORE 18, no. 3 (2022): 287–292, 10.1016/j.explore.2021.01.003. 33541815

[9] S. Kumari and R. Jamwal , “Use of FTIR Spectroscopy Integrated With Multivariate Chemometrics as a Swift, and Non‐Destructive Technique to Detect Various Adulterants in Virgin Coconut Oil: A Comprehensive Review,” Food Chemistry Advances 2 (2023): 100203, 10.1016/j.focha.2023.100203.

[10] P. De Cicco , G. Ercolano , C. Sirignano , et al., “Chamomile Essential Oils Exert Anti‐Inflammatory Effects Involving Human and Murine Macrophages: Evidence to Support a Therapeutic Action,” Journal of Ethnopharmacology 311 (2023): 116391, 10.1016/j.jep.2023.116391. 36948263

[11] “British Pharmacopoeia, Department of Health and Social Security Scottish Home and Health Department,” (1988), Second ed., Office of the British Pharmacopoeia Commission, UK, p. 713.

[12] S. Askin , H. Askin , E. Dursun , et al., “The Hepato‐Renal Protective Potential of Walnut Seed Skin Extract Against Acute Renal Ischemia/Reperfusion Damage,” Cytokine 153 (2022): 155861, 10.1016/j.cyto.2022.155861. 35306426

[13] D. Mehrabani , M. Farjam , B. Geramizadeh , N. Tanideh , M. Amini , and M. R. Panjehshahin , “The Healing Effect of Curcumin on Burn Wounds in Rat,” World Journal of Plastic Surgery 4, no. 1 (2015): 29–35. 25606474 PMC4298862

[14] U. Avsar , Z. Halici , E. Akpinar , et al., “The Effects of Argan Oil in Second‐Degree Burn Wound Healing in Rats,” Ostomy/Wound Management 62, no. 3 (2016): 26–34. 26978857

[15] E. Tiernan and A. Harris , “Butter in the Initial Treatment of Hot Tar Burns,” Burns 19, no. 5 (1993): 437–438, 10.1016/0305-4179(93)90070-o. 8216776

[16] N. Özturk , H. Ceylan , and Y. Demir , “The Hepatoprotective Potential of Tannic Acid Against Doxorubicin‐Induced Hepatotoxicity: Insights Into Its Antioxidative, Anti‐Inflammatory, and Antiapoptotic Mechanisms,” Journal of Biochemical and Molecular Toxicology 38, no. 8 (2024): e23798, 10.1002/jbt.23798. 39108104

[17] F. Guesmi , I. Saidi , R. Abbassi , M. Saidani , N. Hfaiedh , and A. Landoulsi , “Therapeutic Potential of Second Degree's Skin Burns by Topical Dressing of Teucrium Ramosissimum That Promotes Re‐Epithelialization,” Dermatologic Therapy 35, no. 5 (2022): e15428, 10.1111/dth.15428. 35261131

[18] I. Durak , O. Canbolat , M. Kavutçu , H. S. Oztürk , and Z. Yurtarslani , “Activities of Total, Cytoplasmic, and Mitochondrial Superoxide Dismutase Enzymes in Sera and Pleural Fluids From Patients With Lung Cancer,” Journal of Clinical Laboratory Analysis 10, no. 1 (1996): 17–20, 10.1002/(SICI)1098-2825(1996)10:1<17::AID-JCLA4>3.0.CO;2-I. 8926562

[19] H. Zhang , X. Lin , X. Cao , Y. Wang , J. Wang , and Y. Zhao , “Developing Natural Polymers for Skin Wound Healing,” Bioactive Materials 33 (2024): 355–376, 10.1016/j.bioactmat.2023.11.012. 38282639 PMC10818118

[20] Y. Bayir , H. Un , R. A. Ugan , et al., “The Effects of Beeswax, Olive Oil and Butter Impregnated Bandage on Burn Wound Healing,” Burns 45, no. 6 (2019): 1410–1417, 10.1016/j.burns.2018.03.004. 31126777

[21] A. S. Tunç and N. Ercan , “Effect of Topical Sildenafil on Wound Healing and Oxidative Stress in Rats,” Injury 55, no. 6 (2024): 111525, 10.1016/j.injury.2024.111525. 38608450

[22] Y. L. Rao , B. Ganaraja , A. Marathe , et al., “Comparison of Malondialdehyde Levels and Superoxide Dismutase Activity in Resveratrol and Resveratrol/Donepezil Combination Treatment Groups in Alzheimer's Disease Induced Rat Model. 3,” Biotech 11, no. 7 (2021): 329, 10.1007/s13205-021-02879-5. PMC8200337 34189010

[23] V. Singh , M. K. Singh , M. Jain , A. K. Pandey , A. Kumar , and D. K. Sahu , “The Relationship Between BCG Immunotherapy and Oxidative Stress Parameters in Patients With Nonmuscle Invasive Bladder Cancer,” Urologic Oncology 41, no. 12 (2023): e25–e32, 10.1016/j.urolonc.2023.09.008. 37932135

[24] B. M. H. Tammam , O. A. Habotta , M. El‐Khadragy , A. E. Abdel Moneim , and M. S. Abdalla , “Therapeutic Role of Mesenchymal Stem Cells and Platelet‐Rich Plasma on Skin Burn Healing and Rejuvenation: A Focus on Scar Regulation, Oxido‐Inflammatory Stress and Apoptotic Mechanisms,” Heliyon 9, no. 9 (2023): e19452, 10.1016/j.heliyon.2023.e19452. 37662797 PMC10472052

[25] M. Nessler , J. Puchala , F. M. Wood , et al., “Changes in the Plasma Cytokine and Growth Factor Profile are Associated With Impaired Healing in Pediatric Patients Treated With INTEGRA® for Reconstructive Procedures,” Burns 39, no. 4 (2013): 667–673, 10.1016/j.burns.2012.09.001. 23031827

[26] A. M. Kapoun , N. J. Gaspar , Y. Wang , et al., “Transforming Growth Factor‐Beta Receptor Type 1 (TGFbetaRI) Kinase Activity but not p38 Activation is Required for TGFbetaRI‐Induced Myofibroblast Differentiation and Profibrotic Gene Expression,” Molecular Pharmacology 70, no. 2 (2006): 518–531, 10.1124/mol.105.021600. 16707625

[27] H. Ramirez , S. B. Patel , and I. Pastar , “The Role of TGFβ Signaling in Wound Epithelialization,” Advances in Wound Care 3, no. 7 (2014): 482–491, 10.1089/wound.2013.0466. 25032068 PMC4086377

[28] L. Yu , M. C. Hébert , and Y. E. Zhang , “TGF‐Beta Receptor‐Activated p38 MAP Kinase Mediates Smad‐Independent TGF‐Beta Responses,” EMBO Journal 21, no. 14 (2002): 3749–3759, 10.1093/emboj/cdf366. 12110587 PMC126112

[29] S. Schultze‐Mosgau , F. Wehrhan , F. Rödel , et al., “Anti‐TGFbeta1 Antibody for Modulation of Expression of Endogenous Transforming Growth Factor Beta 1 to Prevent Fibrosis After Plastic Surgery in Rats,” British Journal of Oral and Maxillofacial Surgery 42, no. 2 (2004): 112–119, 10.1016/S0266-4356(03)00257-2. 15013542

[30] A. Ghahary , Y. J. Shen , P. G. Scott , Y. Gong , and E. E. Tredget , “Enhanced Expression of mRNA for Transforming Growth Factor‐Beta, Type I and Type III Procollagen in Human Post‐Burn Hypertrophic Scar Tissues,” Journal of Laboratory and Clinical Medicine 122, no. 4 (1993): 465–473. 8228562

[31] L. M. Carrington , J. Albon , I. Anderson , C. Kamma , and M. Boulton , “Differential Regulation of Key Stages in Early Corneal Wound Healing by TGF‐Beta Isoforms and Their Inhibitors,” Investigative Ophthalmology & Visual Science 47, no. 5 (2006): 1886–1894, 10.1167/iovs.05-0635. 16638995

[32] G. Lauer , S. Sollberg , M. Cole , et al., “Expression and Proteolysis of Vascular Endothelial Growth Factor is Increased in Chronic Wounds,” Journal of Investigative Dermatology 115, no. 1 (2000): 12–18, 10.1046/j.1523-1747.2000.00036.x. 10886501

[33] J. Li , Y. P. Zhang , M. Zarei , et al., “A Topical Aqueous Oxygen Emulsion Stimulates Granulation Tissue Formation in a Porcine Second‐Degree Burn Wound,” Burns 41, no. 5 (2015): 1049–1057, 10.1016/j.burns.2014.11.016. 25554261

[34] H. Agarwal , A. Nakara , and V. K. Shanmugam , “Anti‐Inflammatory Mechanism of Various Metal and Metal Oxide Nanoparticles Synthesized Using Plant Extracts: A Review,” Biomedicine & Pharmacotherapy 109 (2019): 2561–2572, 10.1016/j.biopha.2018.11.116. 30551516

[35] J. Jagdeo , E. Kerby , and D. A. Glass, 2nd , “Keloids,” JAMA Dermatology 157, no. 6 (2021): 744, 10.1001/jamadermatol.2020.4705. 33881453

[36] S. A. Eming , T. A. Wynn , and P. Martin , “Inflammation and Metabolism in Tissue Repair and Regeneration,” Science 356, no. 6342 (2017): 1026–1030, 10.1126/science.aam7928. 28596335

[37] M. Cacquevel , N. Lebeurrier , S. Chéenne , and D. Vivien , “Cytokines in Neuroinflammation and Alzheimer's Disease,” Current Drug Targets 5, no. 6 (2004): 529–534, 10.2174/1389450043345308. 15270199

[38] R. A. Elnahas , B. H. Elwakil , S. S. Elshewemi , and Z. A. Olama , “Egyptian Olea Europaea Leaves Bioactive Extract: Antibacterial and Wound Healing Activity in Normal and Diabetic Rats,” Journal of Traditional and Complementary Medicine 11, no. 5 (2021): 427–434, 10.1016/j.jtcme.2021.02.008. 34522637 PMC8427474

[39] M. Chelu , A. M. Musuc , M. Popa , and M. J. Calderon , “Aloe Vera‐Based Hydrogels for Wound Healing: Properties and Therapeutic Effects,” Gels 9, no. 7 (2023): 539, 10.3390/gels9070539. 37504418 PMC10379830

[40] M. A. Chakraborty , “The Antioxidant and Antimicrobial Properties of the Methanolic Extract From Cocos Nucifera Mesocarp,” Food Chemistry 107, no. 3 (2008): 994–999, 10.1016/j.foodchem.2007.08.083.

[41] A. Jamalian , M. Shams‐Ghahfarokhi , K. Jaimand , N. Pashootan , A. Amani , and M. Razzaghi‐Abyaneh , “Chemical Composition and Antifungal Activity of Matricaria Recutita Flower Essential Oil Against Medically Important Dermatophytes and Soil‐Borne Pathogens,” Journal de Mycologie Médicale 22, no. 4 (2012): 308–315, 10.1016/j.mycmed.2012.09.003. 23518164

